# Video capillaroscopy clarifies mechanism of the photoplethysmographic waveform appearance

**DOI:** 10.1038/s41598-017-13552-4

**Published:** 2017-10-16

**Authors:** Mikhail V. Volkov, Nikita B. Margaryants, Andrey V. Potemkin, Maxim A. Volynsky, Igor P. Gurov, Oleg V. Mamontov, Alexei A. Kamshilin

**Affiliations:** 1 ITMO University, Computer Photonics and Videomatics Dept., St. Petersburg, 197101 Russia; 2 Almazov National Medical Research Centre, Dept. of Circulation Physiology, St. Petersburg, 197341 Russia

## Abstract

Photoplethysmography (PPG) devices are widely used in clinical practice but the origin of PPG signal is still under debating. The classical theory assumes that the PPG waveform stems from variations of blood volume in pulsating arteries. In this research we analysed high-speed video recordings of capillaries in a fingernail bed. It was found that speed of erythrocytes in capillaries has pronounced modulation in time, which follows variations of instantaneous blood pressure in arteries. However, the mean speed significantly differs even for neighbour capillaries whereas change of the speed occurs in phase for the most of capillaries. Moreover, the light intensity remitted from the papillary dermis is also modulated at the heartbeat frequency displaying significant correlation with waveforms of the RBC speed. Obtained results can hardly be explained by the classical theory of PPG signal formation. Shallow penetrating visible light acquires modulation of erythrocytes density in the capillary bed without interacting with deeper situated pulsating arteries. Therefore, the capillary bed could serve as a distributed sensor for monitor the status of deep vessels. Better understanding of the photoplethysmography basis will result in a wider range of applications of this fast growing technology in both medical and research practice.

## Introduction

Photoplethysmography (PPG) is an optical method, which is widely used in clinical practice to monitor a patient’s arterial oxygenation and pulse^1^. PPG waveform appears as time-variable intensity of light after its interaction with a live tissue. This phenomenon was first reported in 1936 by Hanzlik *et al*.^2^ but the physiological origin of this modulation is still under debating. Commonly accepted theory assumes that the PPG waveform originates from the relative changes of blood volume in blood vessels, which modulate the light absorption in a tissue^1,3^. Most frequently, the light modulation is observed at the heartbeat frequency displaying clear relationship to the pulsatile vessels. It is known nevertheless that only arteries can change their diameter under varying blood pressure^3^. Study of mechanical properties of capillaries showed that they are noncompliant with minor change of their size due to blood pressure change^4^. It was shown that even terminal arterioles of rabbits do not alter their diameter in response to the stepwise arterial pressure change^5^. However, recent observations of the largest PPG-waveform amplitude at the green light^6–8^ contradict the conventional PPG model. Penetration of green light into the human dermis is rather small (<0.9 mm)^9^, which means that probability of interaction with pulsatile arteries for this light is very small because the arteries are situated deeper than 3 mm below the epidermis^10^. To resolve this contradiction, a new theory of PPG waveform appearance was recently suggested^11^, which hypothesizes that the light modulation arises from compression/decompression of the capillary bed caused by varying transmural pressure in arteries located nearby the region of interest. The new theory assumes that the capillaries themselves are incompressible and do not pulsate at the heartbeat rate but the distance between adjacent capillaries can be readily changed due to stretching/compression of inter-capillary tissue^12^ thus leading to modulation of the capillaries density, and therefore, to intensity modulation of the remitted light.

In this work, we present experimental study of red blood cells (RBC) motion in capillaries assessed by fast video recording of microscopic images of nailfold capillaries synchronously with the electrocardiogram (ECG) recording. Same video capillaroscopy set of images was used for estimation of the PPG waveforms in several finger’s areas close to the nail. Analysis of the experimental data allowed us to shed more light on factors that modulate the PPG signal. Better fundamental understanding of photoplethysmography will result in a wider range of applications of this fast growing technology in both medical and research practice.

## Results

### RBC speed modulation

**Figure 1:**
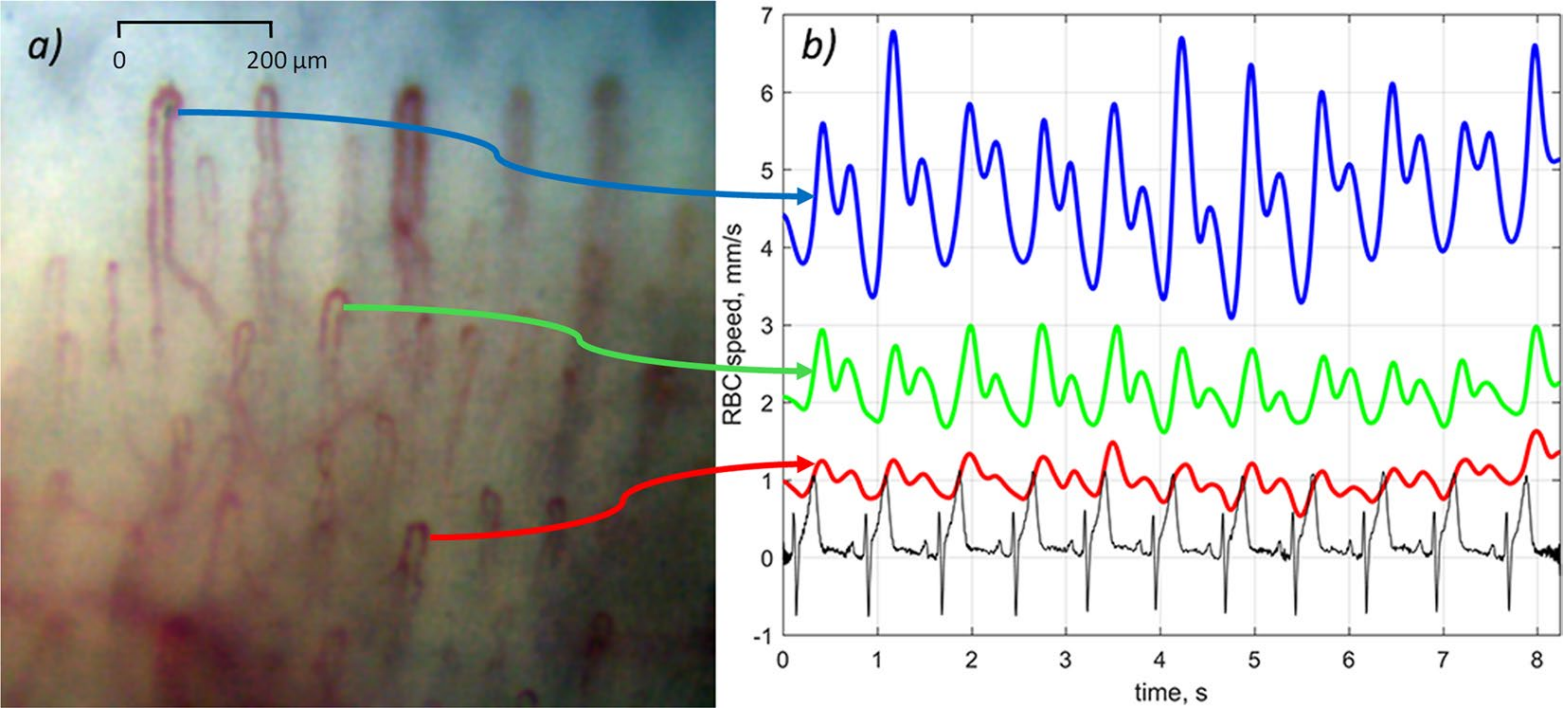
RBC speed modulation in three exemplary capillaries. (**a**) An example of the video capillaroscopy image of finger capillaries. (**b**) RBC speed waveforms (blue, green, and red curves) and simultaneously recorded ECG (black curve). Blue, green, and red arrows connect particular capillaries in which the RBC speed was estimated with the respective graphs.

Typical microscopic image with clearly resolved capillaries is shown in Fig. 1a. The upper part of the image (bluish) corresponds to the nail, whereas the capillary loops in dermis are seen in the lower part. The images were recorded at the rate of 200 frames per second (fps), which allows us to resolve RBCs or their aggregates as well as to evaluate their movement in every capillary. After stabilization of the capillary images, we were able to assess the RBC local speed inside a capillary loop. RBC speed and its dynamics during the experiment was estimated for all capillaries which images were focused in the observation area. Evolution of RBC speed in three exemplary capillaries is shown in Fig. 1b together with ECG. As one can see, the speed is varying in time following the blood pressure modulation in arteries. The shape of these curves resembles classical PPG waveform with anacrotic waves and dicrotic notches^1^. Such a modulation was reported previously^13,14^ but here we underline significant difference of the RBC speed measured in different capillaries. In the capillaries shown in Fig. 1a, the RBC speed varies from 0.5 mm/s to 6.8 mm/s. It is worth noting that the mean speed of RBC is rather high for all studied subjects. The speed averaged over all capillaries for the particular case shown in Fig. 1b is 2.34 mm/s with the standard deviation (STD) of 1.34 mm/s. High STD value underlines significant variation of the RBC speed from one capillary to another. High speed of RBC means that during one second (which is about average duration of the cardiac cycle), erythrocytes move 290 times their own size. To resolve such fast RBC motion, the mentioned above high-speed camera is required.

Seeking for clearness in presenting the graphs in Fig. 1b, only three waveforms of the RBC-speed modulation in three different capillaries are shown. Nonetheless, considering that more than 12 capillaries were resolved in a video frame, the RBC speed was retrieved in much larger number of capillaries. An exemplary set of 12 RBC-speed waveforms is shown in Supplementary Fig. S1. In spite of significant difference of the mean speed in different capillaries, positions of the minima and maxima in the waveform are coinciding for all capillaries. Supplementary Table S1 shows cross-correlation coefficients calculated for 66 combinations of any pair of 12 capillaries. The mean Pierson coefficient in the Table S1 is 0.80 (P < 0.00001) with STD = 0.07. Significant cross-correlation among all RBC-speed waveforms calculated in different capillaries (which are at once visible in a frame) was observed in all participants of the experiment.

### Modulation of reemitted light

**Figure 2:**
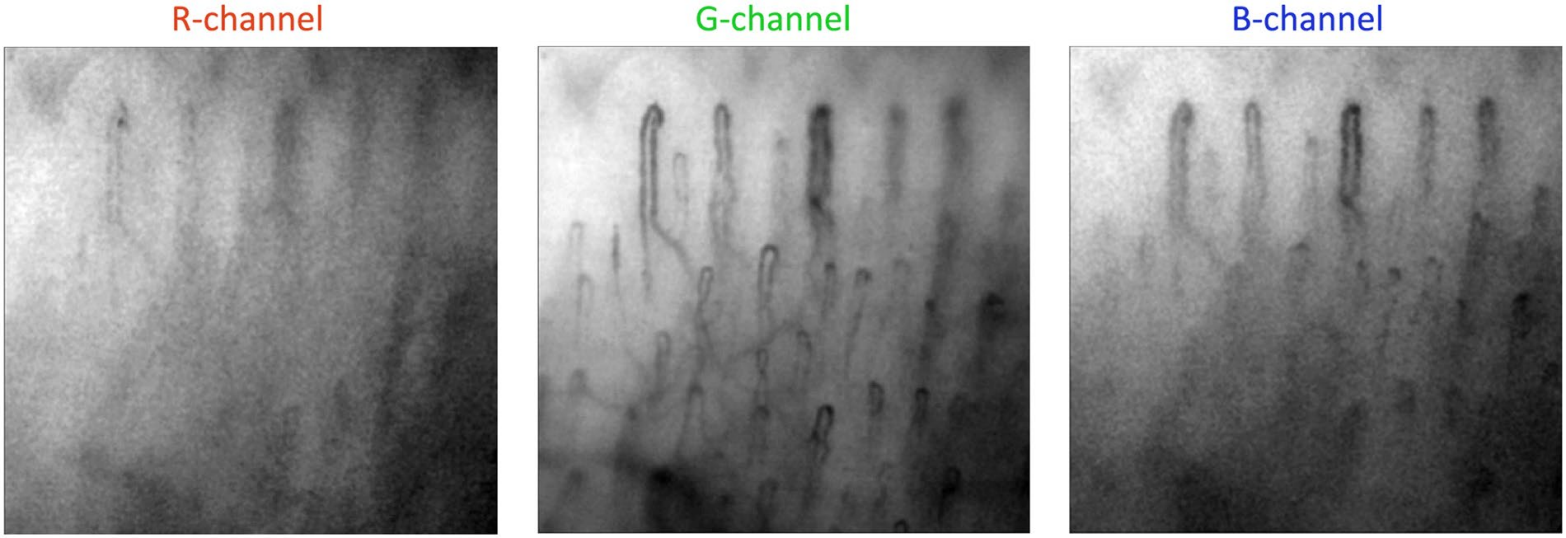
RGB components of the microscopic image of capillaries. The colour version of the image is shown in Fig. 1a.

Microscopic images of capillaries (such as shown in Fig. 1a) were recorded with a digital colour camera under white-light illumination of subject’s finger. The colour frame shown in Fig. 1a was coded as a standard red-green-blue (RGB) image. Figure 2 shows spatial distribution of pixel values separately for each colour channel of the frame presented in Fig. 1a.

One can see that the capillaries are resolved with the best contrast in the green channel, whereas the image in the red channel has the worse contrast. In the blue channel, we resolve only those parts of capillaries, which are situated closer to the epidermis. Observed difference in the image contrast arises from the fact that erythrocytes absorb a light of the blue-green region with seven times higher absorption coefficient than that for the red light^15^. The blue light has smaller penetration depth into a tissue in comparison with the green light because of higher absorption and scattering coefficients^9^. This difference in absorption and scattering coefficients at different wavelengths leads to the highest modulation of green light intensity, and consequently, to the highest pixel value modulation in green channel.

**Figure 3:**
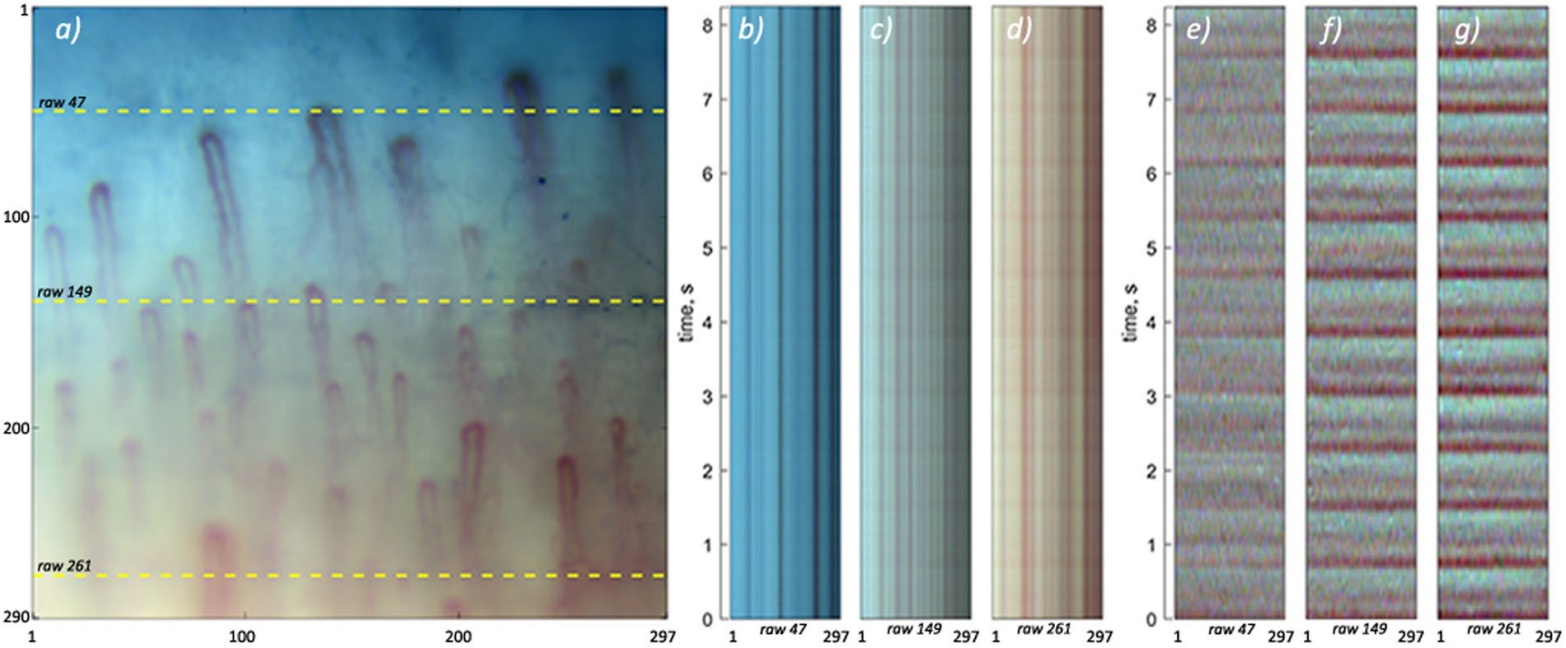
Time-varying modulation of pixel values. (**a**) One of the microscopic images excerpted from the recorded video. (**b**), (**c**), and (**d**) Diagrams showing temporal evolution of pixel values in three rows 47, 149, and 261 marked by dashed lines. (**e**), (**f**), and (**g**) Diagrams with alternating components of the pixel values for the same selected rows.

Dimension of recorded video capillaroscopy images is of 297 × 290 pixels. To analyse the light modulation in different parts of the images at the time scale of heartbeats, we constructed the diagrams in which any single row of pixels from the image frames recorded at different moments is arranged row-by-row in accordance with the current frame number during the time interval of video recording. The number of diagram rows is originated from the total number of recorded frames in the video. These diagrams are shown in Fig. 3b,c and d for the rows 47, 149, and 261, respectively. Row position is shown by dashed line in Fig. 3a.

The diagrams in Fig. 3b–g demonstrate time evolution of remitted light intensity for each selected row of pixels. Dark vertical lines are clearly seen in Fig. 3b–d. Position of each line in the horizontal axis corresponds to a respective capillary horizontal location (see Fig. 3a) with higher light absorption. Any change of the pixel value of a diagram along the vertical direction means its modulation in time. Actually, each diagram in Fig. 3b–d includes a small time-variable component caused by the heartbeats. While the diagram in Fig. 3b (row 47, which is situated near the nail) is almost uniform in time, shiny horizontal areas, which are visible in Fig. 3c and d (rows 149 and 261) under careful observation, suggest occurrence of periodical light intensity modulation. This modulation is poor visible in the figures. To make this modulation more evident, we subtracted a slowly varying component along vertical direction in a diagram separately from each pixel time-variable value thus enhancing an alternating component (AC) of light modulation, which is varying at the heartbeat or higher rate. This modulation at the same rows (47, 149, and 261) is represented in Fig. 3e–g.

Darker horizontal areas are clearly seen in Fig. 3e–g demonstrating modulation of the remitted light with periodicity of about 0.75 s almost uniform along an image row. We will show in the next Section that this modulation well correlates with both the electrocardiogram and RBC speed. Horizontal orientation of the dark lines in the diagrams means synchronous modulation of the pixel values for all pixels in a row. Moreover, these lines appear simultaneously in different rows. Even though only three arbitrarily chosen pixel rows are shown in Fig. 3, the dark horizontal areas in the diagrams appear synchronously in all other rows, as well. It is worth noting that the intensity drops at crossing points of capillaries with rows are disappeared in the diagrams of AC components in Fig. 3e–g. This means independence of temporal reflectivity modulation on the degree of light absorption at an observation point. In all our experiments, we did not find any significant modulation of the capillaries diameter in the time course. All these observations allows us to conclude that the light remitted from the tissue is synchronously modulated in time everywhere in the area of observation irrespectively from the presence of capillary. However, the amplitude of the modulation is smaller in the nail area with lower density of capillaries, whereas it increases in areas with higher capillaries density.

### Photoplethysmographic waveform

**Figure 4:**
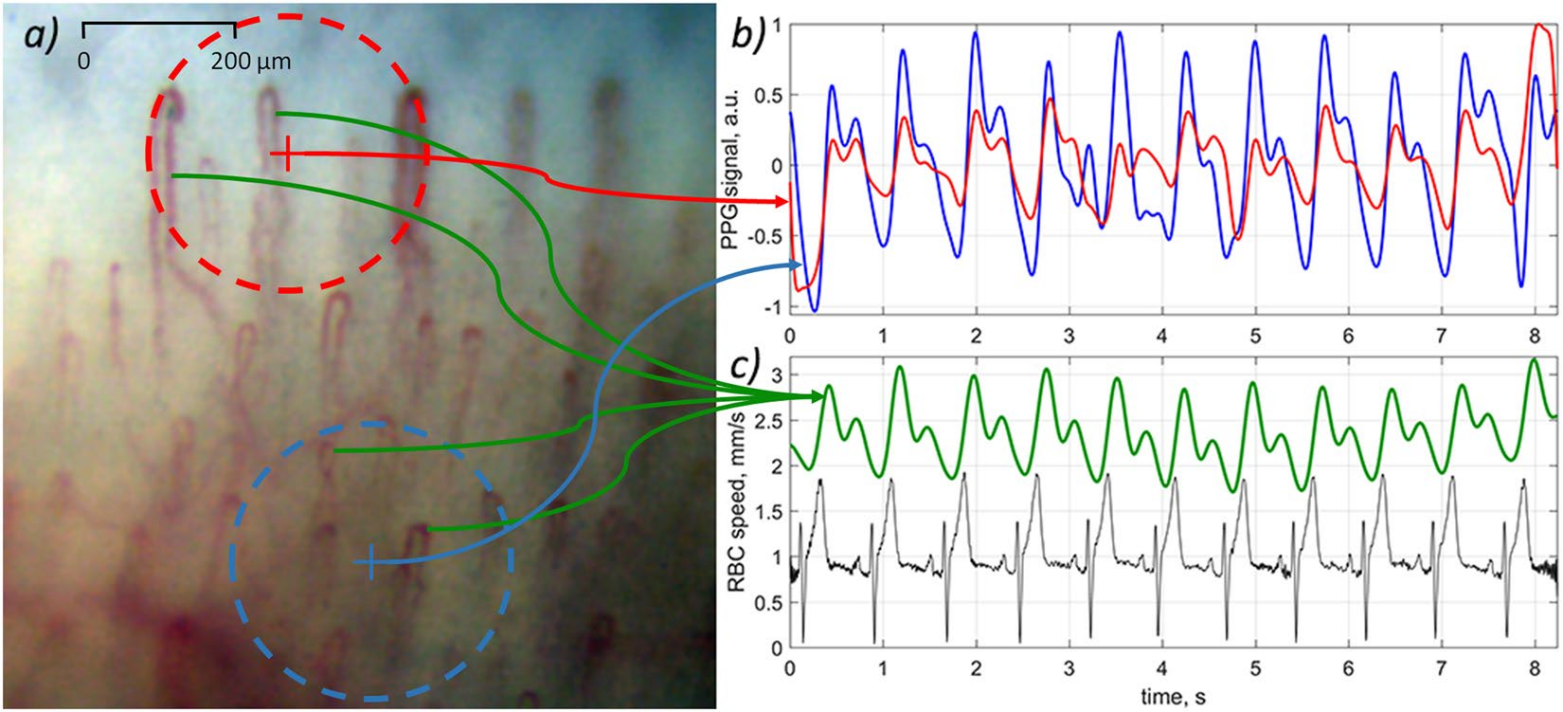
Synchronously recorded PPG waveforms, RBC-speed, and ECG. (**a**) One of the recorded microscopic images of capillaries. (**b**) Two PPG waveforms calculated in the areas marked by the red and blue circles in the image (**a**). (**c**) Mean RBC speed evaluated by video capillaroscopy system averaged over capillaries situated inside the circles (green curve) and ECG signal (black curve). Green arrows connect the graph of RBC speed with four (selected as exemplary) capillaries for which the RBCs speed evolution was first estimated and then averaged.

Synchronous variation of the AC component of all pixels values represents the PPG waveform. Following the contrast difference among the RGB channels shown in Fig. 2, the highest amplitude of the AC component was observed also in the green channel. Two PPG waveforms calculated by averaging of AC components of pixel values over two exemplary areas marked by red and blue circles in Fig. 4a are shown in Fig. 4b. The graphs in Fig. 4b were calculated for pixel values of the green channel only. Note that the diameter of the selected circles is about the size of a single pixel in typical arrangement of imaging photoplethysmography^11,16^, which validates comparison of signals obtained from video capillaroscopy images and from conventional PPG imaging systems. Mean RBC speed calculated for capillaries situated in the selected circles and ECG signal simultaneously recorded with video frames are shown in Fig. 4c. It is seen that the PPG waveform calculated in the blue circle follows the graph of RBC speed, and both the signals are well correlated with ECG. The correlation coefficients of PPG-signal (averaged in the area limited by the blue circle) with ECG and RBC-speed with ECG are 0.82 (P = 0.003) and 0.75 (P = 0.01), respectively. PPG signal from the nail area (red curve in Fig. 4b) has smaller amplitude, and it is more subjected to the noise influence than that originated from the thicker tissue of the dermis (blue curve in Fig. 4b).

As one can see in Fig. 4, the shape of RBC-speed and PPG waveforms are very similar and both correlate with ECG. However, there is also visible difference between these waveforms: PPG is more contaminated by artefacts. Moreover, the amplitude of the PPG waveform calculated over the nail (red circle in the upper part of Fig. 4a) is smaller than that from the lower part (blue circle). Similarity and correlation between the PPG and RBC-speed waveforms were observed for all volunteers participated in our experiments.

**Figure 5:**
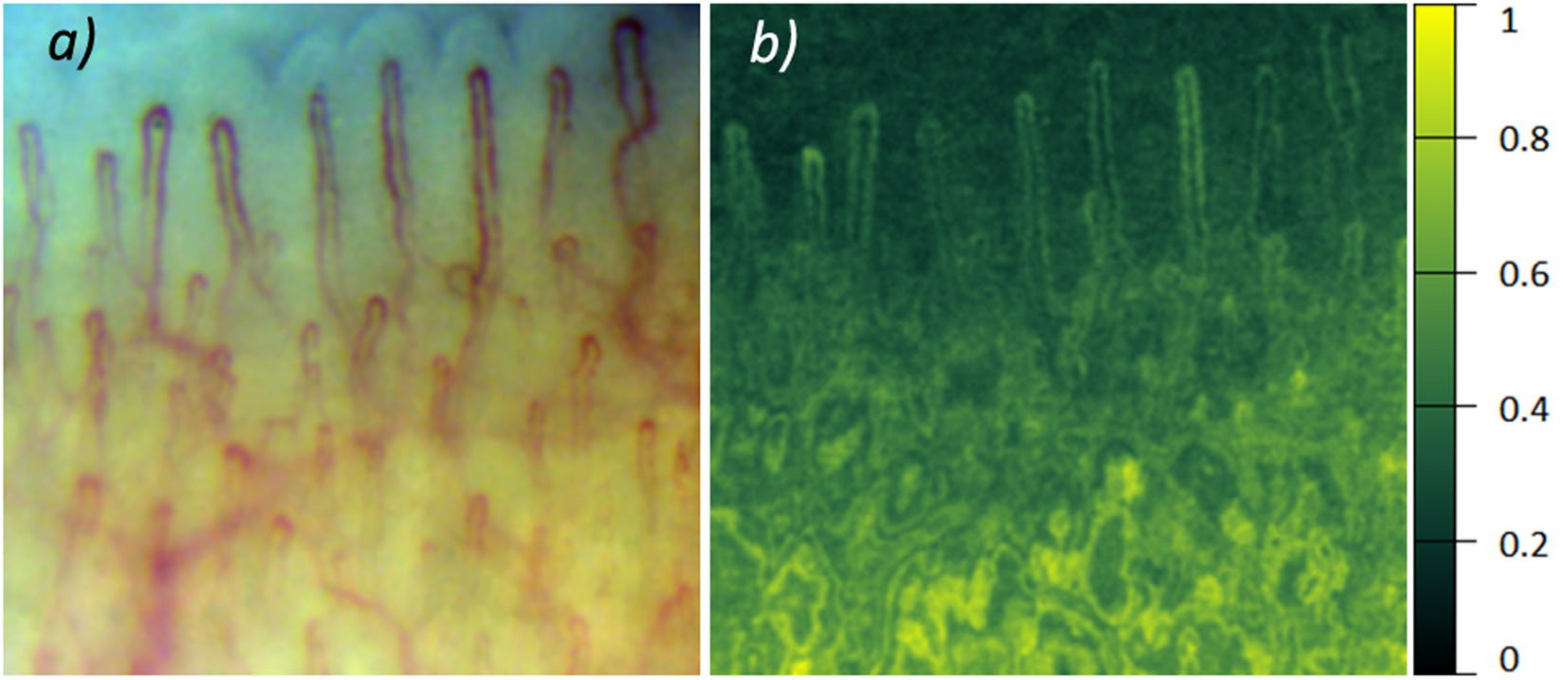
Mapping the amplitude of PPG waveform. (**a**) Microscopic image of capillaries. (**b**) Spatial distribution of the PPG-signal amplitude calculated for the green channel of the image. The color scale in the right side shows the PPG-signal amplitude in arbitrary units.

Spatially resolved distribution of the PPG-signal amplitude is shown in Fig. 5. One can readily distinguish positions of capillaries in the upper part (which corresponds to an area over the nail) of the amplitude map in Fig. 5b whereas the capillaries are hardly distinguishable in the lower part. Due to higher density of capillaries in the lower part of the frame, the light has higher probability of multiple interaction with different capillaries in this part before being reemitted from the tissue. It is worth noting that almost uniform distribution of the PPG signal in the areas of more dense capillaries was also observed for all volunteers. All these observations can hardly find explanation in the frames of conventional PPG model.

## Discussion

It should be noted that the observed speed of erythrocytes in capillaries is so high that it leads to their significant displacement in a capillary during the time scale of heartbeats. Typical frame exposure time accepted for the signal acquisition in imaging PPG systems is about 25 milliseconds^6,8,16^. During this period, the number of erythrocytes in a region of interest is independent from their speed if we assume their density almost constant. Under this assumption, the number of photons absorbed by moving erythrocytes is proportional to the exposure time and mean cross-section of RBCs. Therefore, modulation of RBC speed cannot directly lead to the light intensity modulation. In addition, significant diversity of the RBC speed observed in different capillaries (see Fig. 1) cannot lead to the in-phase modulation of the remitted light intensity in the whole area of observation as it is seen in Fig. 3. Nevertheless, pronounced light-intensity modulation, which correlates well with ECG, was observed everywhere in the area of observation (see Figs 3, 4). This modulation constitutes the PPG waveform, which amplitude is maximal in the green channel, i.e. for shallow penetrating green light which interacts mostly with capillaries, not with other blood vessels. The highest absorption coefficient of RBC at green light^15,17^ explains observed visualization of the capillaries with the highest contrast in the green channel (Fig. 2).

The region of the light-tissue interaction is defined by the processes of light absorption and scattering. It is well known^18^ that the average density of capillaries in human tissue is about 600/mm^3^, which implies a mean separation of 40 µm between adjacent capillaries with their average length of about 1.1 mm, and average diameter of 8.0 µm. The geometrical parameters of capillaries obtained in our video capillaroscopy images are close to these average data. It is haemoglobin absorption, which plays the key role from the viewpoint of the light penetration depth. Blue (460 nm) and green light (530 nm) penetrate the skin up to a depth of 0.7 mm and 0.9 mm, respectively, whereas red (630 nm) light goes deeper to 1.8 mm^9,19^ because of its lower absorption by RBC. Larger absorption of green light provides better distinguishing of RBC from other absorbers thus resulting in higher modulation amplitude at the heartbeat frequency but it decreases the light penetration depth. Incoming light first interacts with the capillaries so that after multiple acts of absorption and scattering, probability of light interaction with deeper situated pulsating arteries and arterioles becomes very small. In other words, the capillary bed serves as efficient screen to prevent direct interaction with large vessels.

We hypothesize that there are at least two mechanisms, which could lead to the observed light-intensity modulation in the capillary bed.

1. Assumption of invariable (during the frame exposure) density of RBC in capillaries may be inapplicable in conditions of strong modulation of their speed, which takes place in all capillaries as it is shown in Fig. 1b and Supplementary Fig. S1. Visual inspection of microscopic video (see Supplementary Video S1) reveals that erythrocytes aggregates continuously change its shape and orientation during propagation via a capillary loop. One may suggest that such variations depend of the RBC speed. Since the speed modulation in different capillaries occurs synchronously in all capillaries (as confirmed by high cross-correlation of RBC-speed waveforms, Supplementary Table S1), the cross-section of light interaction with erythrocytes is modulated in phase in spite of the mean speed difference in neighbour capillaries (Fig. 1b). Synchronous modulation of erythrocytes orientation in different capillaries could be a reason for light intensity modulation. However, the question arises: Is it possible to explain significant increase of the PPG-waveform amplitude by a light contact of the skin with a glass plate^16^ in the frames of this mechanism?
2. According to the alternative theory^11^ of the PPG waveform origin, variations of the transmural pressure in larger and deeper arteries lead to a periodical deformation of the adjacent tissue in the dermis. The deformation changes the density of this tissue, including the density of capillaries in the papillary dermis. The density change affects the blood volume inside the region in which the light efficiently interacts with the tissue regardless of the fact that capillaries are non-compliant^4^. This blood-volume change modulates the incoming light because of high absorption coefficient of haemoglobin. In other words, parameters of light interaction with the capillary bed are modulated via mechanical properties of biological tissue. Muscles contraction, local changes of the lymphatic or venous pressure and external condition (like a contact with a glass) may affect the capillary density in the dermis in addition to the pulsating arterial pressure. It is worth noting that the focusing depth of our microscopic lens was about 50 µm, which is less than one tenth of the green-light penetration depth. Therefore, absence of the observable lateral displacement of capillaries in Fig. 3b–d does not contradict with the hypothesis of inter-capillary distance modulation, including that in the direction of the optical axis.

Additional study is needed for deeper understanding all the details of light interaction with the capillary bed. Nevertheless, our experiments allows us to conclude that varying-in-time pressure of pulsating arteries affects the properties of the capillary bed thus modulating the light reemitted from the live tissue. Therefore, the capillary bed can be considered as a distributed sensor for monitoring the parameters of blood pulsations occurring in the arteries. Consequently, imaging PPG system can be applied for estimation of blood-flow variations in body areas (e.g., in face, extremities, brain cortex) in which such an estimation is difficult to be achieved by other techniques. For example, local diminishing of the pulsation amplitude may indicate not only changes in capillaries but also reduction of blood flow in supplying arteries. Therefore, application of vasoactive either pharmacological or physiological tests could allow semi-quantitative assessment of blood flow and status of the regulation mechanisms.

Moreover, our findings are very promising for development of fundamentally new medical equipment based on imaging photoplethysmography under incoherent illumination. For example, venous occlusion, which results in increase of the blood volume in deep vessels, also changes the PPG signal measured by imaging photoplethysmography, thus representing the speed of blood-volume change^20^.

Since the shape of PPG waveform is defined by variations of arterial blood pressure, imaging PPG system could find important application in appraisal of physiological mechanisms of vascular tone regulation. It can be achieved by accurate measuring the pulse wave delay during it propagation from the heart to the peripheral area under study (pulse transit time)^21^. Changes of this parameter can be caused by sympathetical tone of endothelium, thus allowing study of different regulation mechanisms.

## Methods

### Participants

Eight healthy volunteers (6 males and 2 females, aging 23–36 years) were recruited to participate in the experiment. In total, over 50 capillaroscopy videos were recorded from different fingers of participants. All subjects gave their informed consent of participation in the experiment in the written form. The study was conducted in accordance with the ethical standards laid down in the 1964 Declaration of Helsinki. The study plan was approved on February 6, 2016 (Record No. 30) by the research ethical committee of the Federal Almazov North-West Medical Research Centre prior the experiments.

### Measurement system

**Figure 6:**
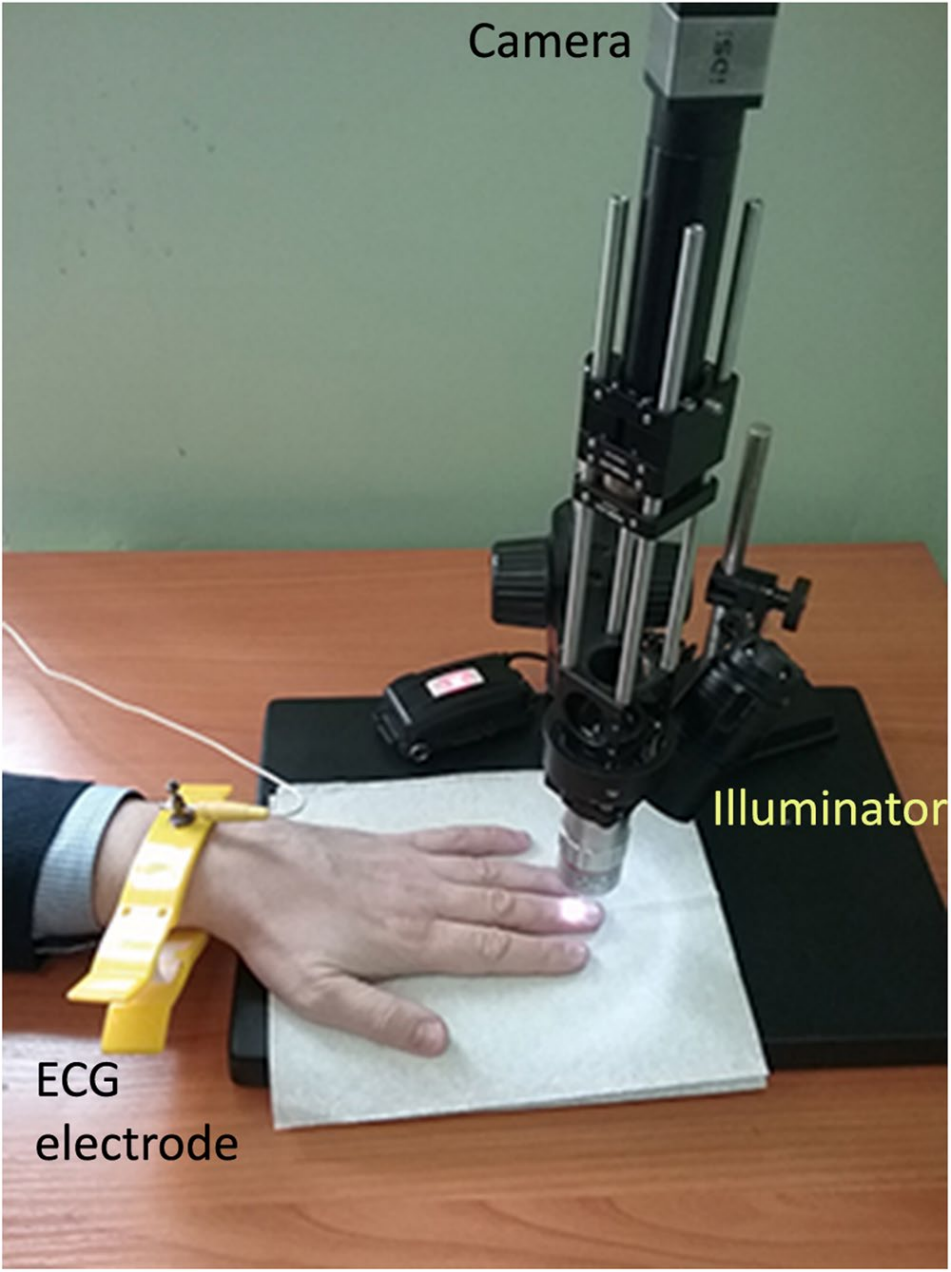
Video capillaroscopy setup for simultaneous recordings of ECG and microscopic video of capillaries.

Layout of the experiment is shown in Fig. 6. An image of nailfold capillaries was magnified by 5× custom-made optical microscope and recorded by a digital colour camera (8-bit model GigE uEye UI-3060CP-C of the Imaging Development Systems GmbH). The microscope lens provided the focusing depth of about 50 μm. The video frames (size of 297 × 290 pixels) were recorded at the rate of 200 fps under illumination of subject’s finger by white light (output power 30 mW, spectral range 400–700 nm). ECG was recorded simultaneously with video by a digital electrocardiograph (model KAP-01-“Kardiotekhnika-EKG” of the Incart Ltd.). Video and ECG recordings were synchronized so that difference between their time scales did not exceed one millisecond. Recorded video frames were saved frame-by-frame in PNG format together with ECG data on a personal computer. All measurements were carried out in a laboratory maintained at a temperature of 22–24 °C. For better adaptation to the laboratory temperature, all participants were asked to sit comfortably at the relaxed position at least 30 min before starting recordings.

### Data processing

The whole set of the recorded data was processed offline by using custom software. The developed software provides high-speed recording of video capillaroscopy images as well as their processing. The main core of the algorithm is space stabilization of video frames using phase correlation. The first processing procedure consists in image stabilization implemented by phase correlation algorithm^22^ applied to adjacent frames and aimed to their accurate spatial matching. As the result, we obtain a video sequence, in which each video camera pixel corresponds to a fixed point in observation field with pixel spacing accuracy through the recorded video frames set. Thereafter, we applied the inter-frame image processing to evaluate the RBC speed. To this end, time-variable areas originated by moving RBCs into capillaries within an image are recognized by comparison of a current image with the averaged one through the total video sequence. The signals originated from the moving RBSs are compared with each other on the matter of mutual inter-frame shift. The shift value is assessed under the criterion of maximal correlation between each pairs of signals belong to adjacent video frames. Obtained values of the shift are averaged over the total video sequence yielding stable estimation of the RBC speed into a capillary as illustrated in Figs 1, 4. It is worth noting the importance of high-speed video recording to resolve moving RBCs and to obtain reliable assessment of their velocity that becomes possible due to noise-immune processing of large enough amount of information containing in a video sequence.

PPG waveforms were calculated in the following steps. First, a frame-by-frame evolution of average pixel values in every chosen regions of interest (such as marked by the circles in Fig. 4) was calculated. Second, a slowly varying component was deduced from the calculated signal providing by high-pass filtering so that the frequencies below 0.3 Hz were removed. Third, by convention used in the photoplethysmography literature^1,3^, the PPG waveforms were inverted in sign so that they positively correlate with varying arterial pressure.

Correlation of the PPG and RBC-speed waveforms with ECG was estimated taking an advantage of the natural heart rate variability. First, we found the temporal position of each R-peak in ECG by using a custom peak-detection algorithm^16^. From these data, duration of each cardiac cycle was evaluated as difference of the time position between two adjacent R-peaks. Second, we detected minima positions in PPG and RBC-speed waveforms in each cardiac cycle by using similar peak-detection algorithm. The time difference between adjacent minima defines the cardiac-cycle duration from PPG and RBC-speed waveforms. As the result, three time series of the cardiac-cycle duration were calculated. Correlation between the series corresponding to PPG and ECG, as well as between RBC-speed and ECG were estimated by using Pearson’s coefficients. Estimation of cross-correlation between waveforms of RBC speed in various pairs of different capillaries was carried out by applying the Pierson’s formula to waveforms with large enough data points in the time scale (total duration of 8.24 s).

## Electronic supplementary material

Dataset 1

Supplementary Video S1.
