# Supplementary material for: Video capillaroscopy clarifies mechanism of the photoplethysmographic waveform appearance
Source: Sci Rep. 2017 Oct 16;7:13298. doi: 10.1038/s41598-017-13552-4 (PMC5643323; doi:10.1038/s41598-017-13552-4)
Supplement: Supplementary file 1 — Dataset 1 [file 41598_2017_13552_MOESM1_ESM.doc]

## **Video capillaroscopy clarifies mechanism of the photoplethysmographic waveform appearance**

*by Mikhail V. Volkov, Nikita B. Margaryants, Andrey V. Potemkin, Maxim A. Volynsky, Igor P. Gurov, Oleg V. Mamontov, and Alexei A. Kamshilin*

## **Supplementary Figure S1.**


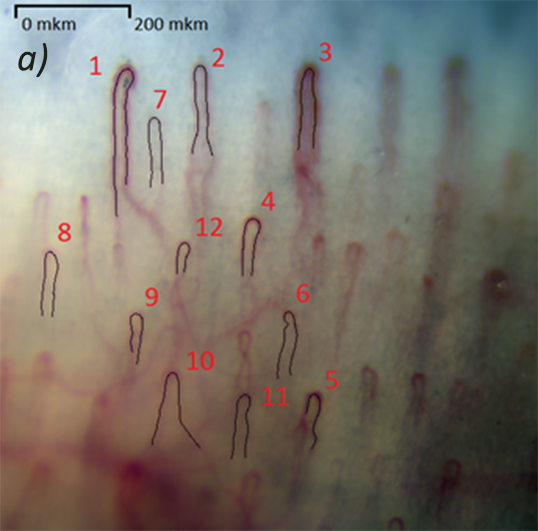


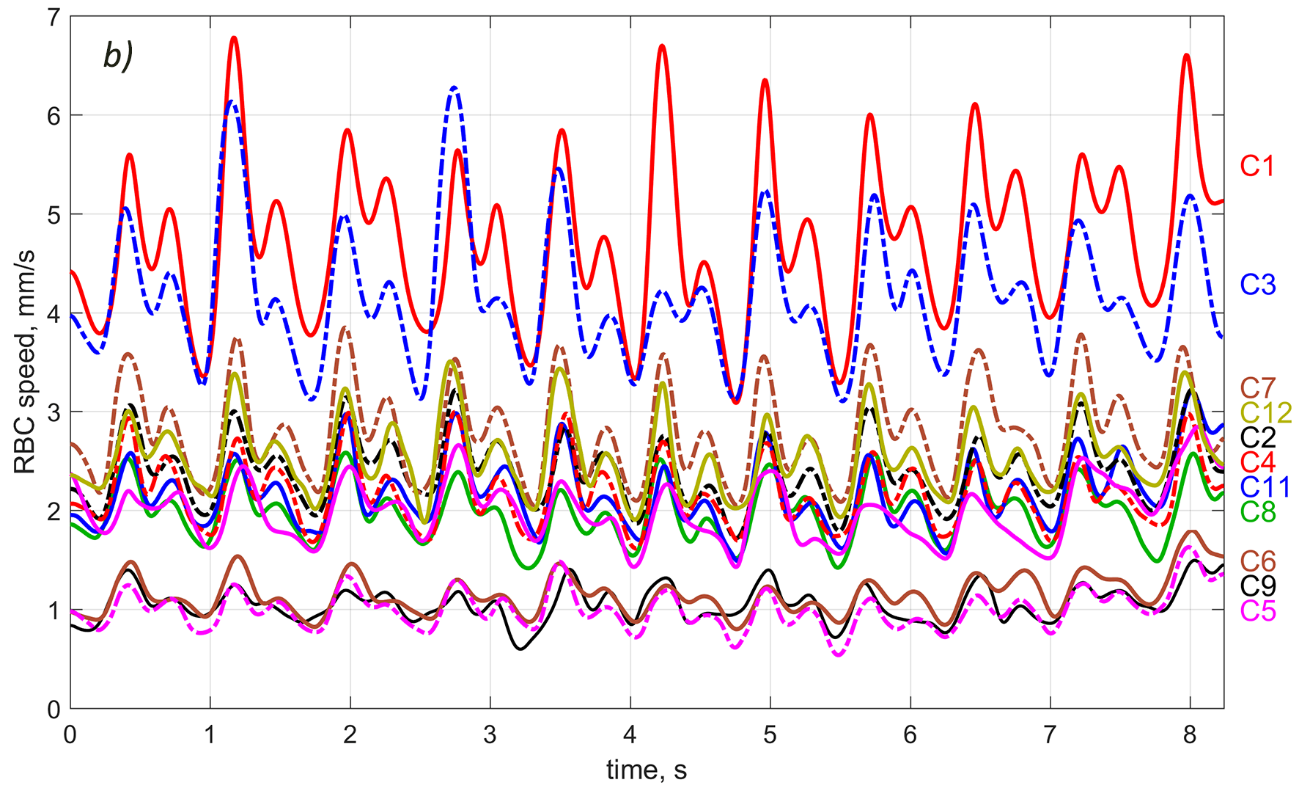


**Figure S1. RBC-speed in different capillaries.** (a) Positions and numbering of 12 chosen capillaries in the video frames for calculation of their RBC speed. (b) Waveforms of RBC-speed in the selected capillaries.

## **Video capillaroscopy clarifies mechanism of the photoplethysmographic waveform appearance**

*by Mikhail V. Volkov, Nikita B. Margaryants, Andrey V. Potemkin, Maxim A. Volynsky, Igor P. Gurov, Oleg V. Mamontov, and Alexei A. Kamshilin*

**Supplementary Table S1.** Pierson’s correlation coefficients of the RBC-speed waveforms calculated for different capillaries. Notations of the capillaries (C1 – C12) are shown in Supplementary Fig. S1*a*.

|  | **C2** | **C3** | **C4** | **C5** | **C6** | **C7** | **C8** | **C9** | **C10** | **C11** | **C12** |
| --- | --- | --- | --- | --- | --- | --- | --- | --- | --- | --- | --- |
| **C1** | 0.87 | 0.78 | 0.86 | 0.84 | 0.81 | 0.91 | 0.90 | 0.76 | 0.70 | 0.81 | 0.86 |
| **C2** |  | 0.81 | 0.90 | 0.85 | 0.80 | 0.90 | 0.88 | 0.73 | 0.77 | 0.88 | 0.90 |
| **C3** |  |  | 0.81 | 0.73 | 0.69 | 0.83 | 0.81 | 0.60 | 0.67 | 0.77 | 0.86 |
| **C4** |  |  |  | 0.85 | 0.76 | 0.89 | 0.87 | 0.73 | 0.73 | 0.85 | 0.89 |
| **C5** |  |  |  |  | 0.90 | 0.82 | 0.76 | 0.77 | 0.84 | 0.92 | 0.74 |
| **C6** |  |  |  |  |  | 0.76 | 0.75 | 0.75 | 0.75 | 0.84 | 0.75 |
| **C7** |  |  |  |  |  |  | 0.89 | 0.71 | 0.70 | 0.81 | 0.90 |
| **C8** |  |  |  |  |  |  |  | 0.78 | 0.68 | 0.78 | 0.84 |
| **C9** |  |  |  |  |  |  |  |  | 0.68 | 0.75 | 0.69 |
| **C10** |  |  |  |  |  |  |  |  |  | 0.85 | 0.72 |
| **C11** |  |  |  |  |  |  |  |  |  |  | 0.84 |
